# Histidine-rich glycoprotein in metabolic dysfunction-associated steatohepatitis-related disease progression and liver carcinogenesis

**DOI:** 10.3389/fimmu.2024.1342404

**Published:** 2024-02-26

**Authors:** Beatrice Foglia, Salvatore Sutti, Stefania Cannito, Chiara Rosso, Marina Maggiora, Alice Casalino, Claudia Bocca, Erica Novo, Francesca Protopapa, Naresh Naik Ramavath, Alessia Provera, Alessandro Gambella, Elisabetta Bugianesi, Frank Tacke, Emanuele Albano, Maurizio Parola

**Affiliations:** ^1^ Department Clinical and Biological Sciences, Unit of Experimental Medicine and Clinical Pathology, University of Torino, Torino, Italy; ^2^ Department Health Sciences and Interdisciplinary Research Centre for Autoimmune Diseases, University Amedeo Avogadro of Eastern Piedmont, Novara, Italy; ^3^ Department Medical Sciences, University of Torino, and Division of Gastroenterology, San Giovanni Hospital, Torino, Italy; ^4^ Department of Pediatrics, School of Medicine, Washington University, St Louis, MO, United States; ^5^ Department of Hepatology and Gastroenterology, Charité-Universitatsmedizin Berlin, Berlin, Germany

**Keywords:** MASH, histidine-rich glycoprotein, inflammation, fibrogenesis, liver carcinogenesis, HCC, hepatocellular carcinoma, chronic liver diseases

## Abstract

**Background:**

Metabolic dysfunction-associated steatotic liver disease (MASLD), previously non-alcoholic fatty liver disease (NAFLD), is a leading cause of chronic liver disease worldwide. In 20%–30% of MASLD patients, the disease progresses to metabolic dysfunction-associated steatohepatitis (MASH, previously NASH) which can lead to fibrosis/cirrhosis, liver failure as well as hepatocellular carcinoma (HCC). Here we investigated the role of histidine-rich glycoprotein (HRG), a plasma protein produced by hepatocytes, in MASLD/MASH progression and HCC development.

**Methods:**

The role of HRG was investigated by morphological, cellular, and molecular biology approaches in (a) HRG *knock-out* mice (HRG^−/−^ mice) fed on a CDAA dietary protocol or a MASH related diethyl-nitrosamine/CDAA protocol of hepatocarcinogenesis, (b) THP1 monocytic cells treated with purified HRG, and (c) well-characterized cohorts of MASLD patients with or without HCC.

**Results:**

In non-neoplastic settings, murine and clinical data indicate that HRG increases significantly in parallel with disease progression. In particular, in MASLD/MASH patients, higher levels of HRG plasma levels were detected in subjects with extensive fibrosis/cirrhosis. When submitted to the pro-carcinogenic protocol, HRG^−/−^ mice showed a significant decrease in the volume and number of HCC nodules in relation to decreased infiltration of macrophages producing pro-inflammatory mediators, including IL-1β, IL-6, IL-12, IL-10, and VEGF as well as impaired angiogenesis. The histopathological analysis (H-score) of MASH-related HCC indicate that the higher HRG positivity in peritumoral tissue significantly correlates with a lower overall patient survival and an increased recurrence. Moreover, a significant increase in HRG plasma levels was detected in cirrhotic (F4) patients and in patients carrying HCC vs. F0/F1 patients.

**Conclusion:**

Murine and clinical data indicate that HRG plays a significant role in MASLD/MASH progression to HCC by supporting a specific population of tumor-associated macrophages with pro-inflammatory response and pro-angiogenetic capabilities which critically support cancer cell survival. Furthermore, our data suggest HRG as a possible prognostic predictor in HCC patients with MASLD/MASH-related HCCs.

## Introduction

Metabolic dysfunction-associated steatotic liver disease (MASLD) is the novel acronym for a chronic liver disease (CLD) associated with at least one cardiovascular risk (1), previously known as non-alcoholic fatty liver disease or NAFLD, which is widely acknowledged as the emerging leading cause of CLD worldwide (2–6). MASLD is estimated to affect approximately 1 billion of individuals and to have a global prevalence in the general population of approximately 25% (4–6), which is predicted to further increase by 2030 in USA, Europe, and Southeast Asia (7). MASLD is mostly diagnosed in obese and type 2 diabetes (T2D) patients and is currently viewed as the hepatic manifestation of the metabolic syndrome (8–12). MASLD encompasses a spectrum of conditions ranging from simple steatosis to the progressive form of metabolic dysfunction-associated steatohepatitis (MASH, formerly referred to as non-alcoholic steatohepatitis or NASH). Simple steatosis, detected in the large majority (75%–80%) of patients, is usually a benign condition, not progressive or slowly progressive (10, 13). The diagnosis of MASH applies to the remaining patients and requires histopathological evidence of liver steatosis associated with aspects of parenchymal injury such as ballooning, apoptosis, focal necrosis, lobular and/or portal inflammatory infiltrate, and a variable degree of fibrosis. MASH patients are at a significant risk to progress towards a more advanced stage of CLD, with approximately 15%–20% of MASH patients developing cirrhosis over three to four decades (5, 13–17).

Interestingly, insulin resistance and obesity, leading to chronic hepatic inflammation and lipid dysmetabolism in MASH patients, can also promote a pro-carcinogenic profile and the development of hepatocellular carcinoma (HCC) (18, 19), the sixth most diagnosed cancer and the third leading cause of cancer-related death worldwide (20). MASH-related HCC is recognized as the most rapidly growing cause of HCC in liver transplant candidates in the USA (19, 21). At present, the mechanisms underpinning MASLD progression are still largely unknown, and this reflects a lack of validated therapeutic strategies and of reliable biomarkers able to predict the individual risk of HCC development and clinical outcome. Such a situation is relevant since MASH-related HCC is usually diagnosed at a later stage and, unlike other etiologies, can develop also in non-cirrhotic patients, usually asymptomatic or pauci-symptomatic for which no reliable HCC screening protocol is currently available (4–6, 22).

HCC development and progression are known to proceed through a multi-step accumulation of genetic and epigenetic alterations, eventually leading to a remarkable tumor heterogeneity, in terms of phenotype and functional signatures (23). This makes difficult to unveil major disease mechanisms, to reach an early diagnosis, and to choose the most efficient therapeutic approach. Moreover, to emphasize the relevance of tumor–stroma interactions, a critical role in HCC development and progression is played by fibrogenesis, angiogenesis, and inflammation (6, 23, 24) which are critical also for MASH-related HCC (25–28).

The present study focuses on histidine-rich glycoprotein (HRG), a 75-kDa heparin-binding protein expressed by hepatocytes and considered as a negative acute-phase response protein (29–31). Due to its peculiar multi-domain structure, HRG can interact with different ligands, including heme, thrombospondin-1 (TSP1), plasmin/plasminogen, complement C1q, fibrinogen, and immunoglobulin G (29–31). Accordingly, HRG can modulate critical biological processes including blood coagulation and fibrinolysis, angiogenesis, and anti-tumor immune response as well as the clearance of immune-complexes and dead cells (29–33). Pertinent to the present study, HRG has been proposed to act as a potent activator of macrophages, leading to their polarization into a pro-inflammatory phenotype (34, 35). In particular, the use of HRG *knock-out* (HRG^-/-^) mice has revealed that HRG can promote experimental chronic liver injury and fibrosis progression (35). The latter study also showed that MASH patients significantly upregulated hepatocellular HRG expression as an event associated with pro-inflammatory polarization of macrophages (35). More recently, HRG has been shown to be expressed by hepatocytes as a hypoxia-inducible factor (HIF)2α-dependent mediator, with HIF2α activation contributing to murine and human MASLD progression through HRG upregulation in hepatocytes (36).

At present, only few studies have investigated HRG’s role in cancer development and, to our knowledge, its role in MASH-related hepatocarcinogenesis has never been addressed. Moreover, differently from what is outlined by experimental and clinical data that strongly suggests HRG as a positive contributor to CLD progression (34–36), the few available evidences concerning the role of HRG in carcinogenesis suggests instead HRG as a putative antitumor mediator—for instance, HRG deletion results in enhanced tumor growth and metastatic capacity in mice transplanted with fibrosarcoma or pancreatic carcinoma cells in relation to HRG’s capacity of promoting vessel abnormalities and suppressing antitumor immune responses (37). On the same vein, *in vitro* and *in vivo* studies have shown that HRG overexpression in hepatoma cell lines led to a decrease in cell proliferation, colony-forming ability, and tumor growth along with increased cancer cell apoptosis (38). Furthermore, HRG has been proposed to suppress HCC cell growth by inducing cancer cell apoptosis, possibly though an interaction of TNFα with its type 1 receptor (39). The only available data concerning human HCC were obtained from few cases of liver cancer or induced by aflatoxin B1 or with unspecified etiology (38, 40, 41), reporting a decrease in HRG expression in tumor tissue compared to non-tumoral tissue.

The present study was designed to elucidate the effective role of HRG in relation to MASH-related liver carcinogenesis. The role of HRG was investigated *in vivo* by morphological, cellular, and molecular biology approaches in MASLD/MASH patients with or without HCC as well as in HRG^-/-^ mice submitted to a MASH-related protocol of liver carcinogenesis. Additional data were obtained by exposing *in vitro* macrophages and liver myofibroblasts to purified HRG.

## Materials and methods

### Materials

The enhanced chemiluminescence (ECL) reagents and nitrocellulose membranes were from BioRad company (Hercules, CA, USA), The following antibodies were used: anti-GAPDH (sc-20357), anti-caspase3 (sc7148), anti-YAP (sc-15407), anti-c-MYC (sc-788), anti-vinculin (sc-73614), anti-VEGF-A (sc152), anti-PECAM1 (sc1506) from Santa Cruz Biotechnology (Dallas, Texas, USA); anti-β-actin (A5441), anti-HRG (HPA050269), and anti-α-SMA (A2547) from Merck KGaA (Darmstadt, Germany); anti-PCNA (PA5-27214), anti-VE-cadherin (PA5-19612), anti-Cd105 (PA5-12511), and anti-HRG (PA5-76389) from ThermoFisher Scientific (Rockford, IL, USA); anti-cleaved caspase-3 (#9661) from Cell Signaling Technology (Danvers, MA, USA); anti-F4/80 (14–4801–82) from Ebioscience (CA, USA); and anti-HRG (AF1905) from R&D systems (Minneapolis, MN, USA). Mayer’s hematoxylin, Trizol®, and all primers used in quantitative real-time PCR (qPCR) reactions were purchased from Merck KGaA (Darmstadt, Germany). Prof. Wilhelm Jahnen-Dechent, (Helmholtz Institute for Biomedical Engineering, RWTH University-Hospital Aachen, Aachen, Germany) provided the HRG^-/-^ mice (35). The qPCR analyses were carried out using the MiniOpticonTM Real-Time PCR Detection System instrument of the BioRad company (Hercules, CA, USA), which also supplied the EvaGreen master mix reagent. Applied Biosystems (Foster City, CA, USA) supplied the High-Capacity cDNA Reverse Transcription Kit for reverse transcription. The anti-mouse and anti-rabbit of the EnVision System-HRP Labeled Polymer (Dako-Agilent, Santa Clara, USA) and the secondary goat anti-rat antibody (Abcam, Cambridge, UK) were used as secondary antibodies. The company Laboratorio Dottori Piccioni (Gessate, Italy) provided the CDAA diet. The 8-week-old male C57BL/6 wild-type mice were purchased from Charles River Laboratories (Charles River UK Ltd., Margate, UK).

### Animal experimentation

For the study of MASLD/MASH-associated HCC, an experimental protocol of hepatic carcinogenesis was used using mice carrying the constitutive deletion of HRG described and characterized in a previous study (35) and related wild-type mice. These knockout mice (HRG^-/-^) were kindly provided by Prof. Wilhelm Jahnen-Dechent (Helmholtz Institute for Biomedical Engineering, RWTH University-Hospital Aachen, Aachen, Germany) (35). MASLD-associated liver carcinogenesis was induced in male HRG^-/-^ mice (*n* = 11) and related control wild-type sibling littermates (WT, *n* = 12), with an established experimental protocol involving a single administration of diethyl nitrosamine (DEN; 25 mg/kg bw, i.p.) at the age of 2 weeks followed by feeding with a choline-deficient L-amino acid (CDAA)-defined diet (Laboratorio Dottori Piccioni, Gessate, Italy) for 25 weeks starting from the age of 6 weeks (42). The nutritional content of CDAA diet includes 435.1 kcal/100 g, 55.7% carbohydrates, 13.2% proteins, and 31% lipids. Analysis was performed in two neoplastic nodules obtained from any experimental animal. In preliminary experiments, 8-week-old male control wild-type mice (WT, *n* = 6) and HRG *knock-out* mice (HRG^–/–^, *n* = 9) were fed with the CDAA lipogenic diet for 24 weeks to mimic the MASLD–MASH progression. The animal experiments complied with national ethical guidelines for animal experimentation, and the experimental protocols were approved by the Italian Ministry of Health.

### Biochemical analyses

Plasma alanine aminotransferase (ALT) was determined by using spectrometric kits supplied by Radim S.p.A. (Pomezia, Italy). Circulating HRG was evaluated by using commercial ELISA kits (EH243RB) supplied by ThermoFisher Scientific (Rockford, IL, USA). The tissue triglyceride concentration was determined by using Triglyceride Assay Kit—Colorimetric (MAES0165) supplied by AssayGenie (Dublin, Ireland).

### Patients and samples

The analysis conducted on MASLD patients was approved by the ethics committee of the Azienda Ospedaliera Universitaria Città della Salute, Torino, Italy. For IHC analysis, we analyzed liver specimens from MASLD patients (*n* = 27) carrying HCC and referred to the Division of Gastro-Hepatology of the University of Turin. All samples were collected at the time of resection or transplantation. The second cohort of patients (*n* = 78) included in the study underwent venous blood sampling at the time of diagnosis. EDTA plasma was collected and stored at −80°C until analysis. Fibrosis was staged F0 to F4 and classified as absent (F0), mild (F1–F2), and severe (F3–F4). MASH was defined by the local pathologist according to the combined presence of steatosis, hepatocyte ballooning, and lobular inflammation with or without fibrosis. The clinical and biochemical features of the two cohorts are reported in **Tables 1** and **2**, respectively. All subjects gave informed consent to the analysis and the study protocol, conformed to the ethical guidelines of the 1975 Declaration of Helsinki, and planned according to the guidelines of the local ethical committee.

**Table 1:** Clinical and biochemical characterization of MASLD/MASH patients carrying HCC related to the histology cohort.

Variable	Histology cohort
Number of patients (male/female)	27 (25/2)
Age (years)	71 (49–86)
BMI (kg/m^2^)	28.2 (22.3–34.6)
T2DM	85.2%
AST (U/I)	37 (17–83)
ALT (U/I)	37 (13–86)
γ-GT (U/I)	111 (14–307)
Albumin (g/L)	4.1 (3.3–4.8)
Total bilirubin (mg/dL)	0.9 (0.3–2.5)
HDL (mg/dL) <40 m/<50 f	61.1%/60.0%
Triglycerides (mg/dL)	111 (77–155)
Edmondson–Steiner grading	G1: 0% G2: 74.1% G3: 18.5% G4: 7.4%
Number of nodules	1 (1–4)

**Table 2:** Clinical and biochemical characteristics of MASLD-MASH and HCC patients related to the plasma cohort.

Variable	All	F0/1	F3	Cirrhosis	HCC
Number of patients (male/female)	78 (54/24)	10 (7/3)	6 (3/3)	19 (8/11)	43 (36/7)
Age (years)	63 (20–84)	40 (29–63)	55 (20–63)	58 (31–70)	66 (53–84)
BMI (kg/m^2^)	28.7 (21.2–45.7)	26.5 (22.1–36.9)	28.85 (23.4–32.3)	28.9 (24.2–45.7)	29.1 (21.2–40.8)
T2DM	52.6%	20.0%	0%	73.7%	58.5%
AST (U/I)	35 (16–273)	32 (16–58)	49 (25–77)	32 (18–273)	35.5 (18–248)
ALT (U/I)	39 (10–273)	51 (14–94)	62 (35–154)	44 (22–273)	36.5 (10–152)
γ-GT (U/I)	91 (15–554)	79.5 (15–197)	47 (28–317)	77 (16–482)	93 (22–554)
Albumin (g/L)	4.0 (2.4–5.3)	4.4 (4.0–5.3)	4.35 (3.9–4.6)	4.2 (3.9–4.6)	3.7 (2.4–4.7)
Total bilirubin (mg/dL)	0.8 (0.3–18.2)	0.7 (0.3–0.9)	0.9 (0.5–1.6)	0.6 (0.3–1.5)	1.2 (0.4–18.2)
Total cholesterol (mg/dL)	166.5 (80–314)	207 (162–314)	192 (165–256)	161 (130–267)	165 (80–267)
Triglycerides (mg/dL)	108 (64–812)	156 (98–441)	100 (66–159)	120 (67–812)	101 (64–275)
Child score				A 100%	
BCLC					0: 11.6% A: 48.8% B: 20.9% C: 14.0% D: 4.7%
Number of nodules					2 (1–7)

### TCGA analysis

The TCGA database was accessed on 29.09.2023 through the GEPIA webserver (http://gepia.cancer-pku.cn/index.html) to retrieve HRG mRNA expression data, available for 364 patients, in the Liver Hepatocellular Carcinoma cohort (LIHC). Survival and disease-free survival analysis were run through the GEPIA webserver using the following cutoff values: high-HRG = 50%, low-HRG = 50%. A comparison of tumor (*n* = 369) vs. normal (*n* = 50) tissue was assessed using log2(TPM + 1) transformed the expression data for plotting. The dataset selected were “TCGA tumors” vs. “TCGA normal”. The method for differential analysis is one-way ANOVA, using disease state (tumor or normal) as the variable for calculating differential expression. The *p*-value cutoff selected was 0.0001.

### Cell lines and culture conditions

The *in vitro* experiments described in the present study were performed on human monocytes of the THP-1 cell line, acquired from the American Type Culture Collection (ATCC, Manassas, VA 20108 USA). The THP-1 cell line was differentiated into macrophages by treatment for 48 h with phorbol 12-myristate 13-acetate (PMA, 50 nM). The THP-1 cells were cultured in RPMI medium containing 10% fetal bovine serum, 100 U/ml of penicillin, 100 mg/ml of streptomycin, and 25 mg/ml of amphotericin-B (Merck Life Science, Milan, Italy). The differentiated THP-1 cells, after 24 h of incubation with fresh culture medium, were stimulated with 80 µg/ml purified human HRG kindly provided by Prof. Wilhelm Jahnen-Dechent (Helmholtz Institute for Biomedical Engineering, RWTH University-Hospital Aachen, Aachen, Germany) (35) at different time points.

### Quantitative real-time PCR

RNA extraction, complementary DNA synthesis, and quantitative real-time PCR (qPCR) reactions were performed on cell samples or murine liver specimens as previously described (36, 42). The mRNA levels were measured by qPCR, using the SYBR® green method as described (36, 42). More details and oligonucleotide sequences of primers used for q-PCR are presented in **Table 3**.

**Table 3:** Oligonucleotide sequences of the primers used for qPCR.

Gene	Primers
ATGL (mouse)	(FW) 5′ CTCACATCTACGGAGCCTCG 3′ (RV) 5′ CGGATGGTCTTCACCAGGTT 3′
CD36 (mouse)	(FW) 5′ TGCTGGAGCTGTTATTGGTG 3′ (RV) 5′ TGGGTTTTGCACATCAAAGA 3′
CPT1A (mouse)	(FW) 5′ CCAGGCTACAGTGGGACATT 3′ (RV) 5′ GAACTTGCCCCATGTCCTTGT 3′
F4/80 (mouse)	(FW) 5′ GTACAGATGGGGGATGACCAC 3′ (RV) 5′ GACTGAGTTAGGACCACAAGGTGAG 3′
FABP4 (mouse)	(FW) 5′ CATCAGCGTAAATGGGGATT 3′ (RV) 5′ TCGACTTTCCATCCCACTTC 3′
FASN (mouse)	(FW) 5′ TGGGTTCTAGCCAGCAGAGT 3′ (RV) 5′ ACCACCAGAGACCGTTATGC 3′
HMGCL (mouse)	(FW) 5′ GGCTTGACGTCCCTCCG 3′ (RV) 5′ GGAGAAACAAAGCTGGTGGC 3′
HMGCS2 (mouse)	(FW) 5′ GAGCGATGCAGGAAACTTCG 3′ (RV) 5′ GTATCTGTTTTGGCCAGGGGA 3′
HRG (mouse)	(FW) 5′ CCACCACATGGACACTCAAG 3′ (RV) 5′ GCAATTTGGCAAAGAGAAGC 3′
IL-6 (mouse)	(FW) 5′ CTGATGCTGGTGACAACCAC 3′ (RV) 5′ TCCACGATTTCCCAGAGAAC 3′
IL-12 (mouse)	(FW) 5′ CTTTGATGATGACCCTGTGC 3′ (RV) 5′ GATTCTGAAGTGCTGCGTTG 3′
IL-1β (mouse)	(FW) 5′GAAATGCCACCTTTTGACA 3′ (RV) 5′TTGGAAGCAGCCCTTCATCTT 3′
IL-10 (mouse)	(FW) 5′ TTTCACAGGGGAGAAATCG 3′ (RV) 5′AGGAACCTGAAACTCCCCAG 3′
IL23A (mouse)	(FW) 5′ ACCAGCGGGACATATGAATC 3′ (RV) 5′ GGATCCTTTGCAAGCAGAAC 3′
Ki67 (mouse)	(FW) 5′ CATGCAAACCCTCACACTTG 3′ (RV) 5′GCTGGTTCCAATTTCTGAGC 3′
MCAD (mouse)	(FW) 5′ GAAGCCACGAAGTTCAACC 3′ (RV) 5′ TGAGCCTAGCGAGTTCAACC 3′
MMP1 (mouse)	(FW) 5′ AGTTGACAGGCTCCGAGAAA 3′ (RV) 5′GGCACTCCACATCTTGGTTT 3′
MMP2 (mouse)	(FW) 5′ ACCCTGGGAGAAGGACAAGT 3′ (RV) 5′ ATCACTGCGACCAGTGTCTG 3′
PDL-1 (mouse)	(FW) 5′ GAGATCACAGCCAGGGCAAA 3′ (RV) 5′ TGTGGAGGATGTGTTGCAGG 3′
α-SMA (mouse)	(FW) 5′ GCCTCTTCCTGACAAACGAG 3′ (RV) 5′ TGACTGCCGAAACATCTCTG 3′
SREBP1 (mouse)	(FW) 5′ GATCAAAGAGGAGCCAGTGC 3′ (RV) 5′ TAGATGGTGGCTGCTGAGTG 3′
TBP (mouse)	(FW) 5′ CACATCACAGCTCCCCACCA 3′ (RV) 5′ AGCGGAGAAGATGCTGGAAAC 3′
TGFβ (mouse)	(FW) 5′ GGACTCTCCACCTGCAAGAC 3′ (RV) 5′ GACTGGCGAGCCTTAGTTTG 3′
TREM2 (mouse)	(FW) 5′ CCTGCAGAAAGTACTGGTGGA 3′ (RV) 5′ TCTCTTGATTCCTGGAGGTGC 3′
TIMP2 (mouse)	(FW) 5′ GCATCACCCAGAAGAAGAGC 3′ (RV) 5′ GGGTCCTCGATGTCAAGAAA 3′

### Western blot analysis

Total cell/tissue lysates, obtained as previously described (36, 42), were subjected to sodium dodecyl sulfate–polyacrylamide gel electrophoresis on 12%, 10%, or 7.5% acrylamide gels, incubated with the desired primary antibodies, then with peroxidase-conjugated anti-mouse or anti-rabbit immunoglobulins in Tris-buffered saline-Tween containing 2% (w/v) non-fat dry milk, and finally developed with the ECL reagents according to manufacturer’s instructions. Sample loading was evaluated by reblotting the same membrane with antibodies raised against GAPDH, β-actin, or vinculin.

### Immunohistochemistry

The paraffin-embedded human liver specimens and/or murine liver specimens used in this study were immuno-stained as previously reported (36, 42). Briefly, paraffin sections (4 μm in thickness), mounted on poly-L-lysine-coated slides, were incubated with the antibodies against HRG (mice samples dil. 1:300 v/v, human samples 1:50 v/v), F4/80 (dil. 1:500 v/v), and α-SMA (dil. 1:2000, v/v). After blocking endogenous peroxidase activity with 3% hydrogen peroxide and performing microwave antigen retrieval, primary antibodies were labeled by using EnVision, HRP-labeled System (DAKO), and visualized by using 3′-diaminobenzidine substrate. Collagen deposition was evidenced by Picro-Sirius Red staining as previously described (36), and quantification of fibrosis in the murine liver was performed by histo-morphometric analysis using a digital camera and a bright field microscope to collect images that were then analyzed by employing the ImageJ software (**Supplementary Figure S1**) as described by AR Crowe and W Yue (43). HRG immunohistochemical stain was evaluated by a pathologist and scored semi-quantitatively according to the H-score. Briefly, HRG expression was scored by combining the intensity of staining and the percentage of cells expressing the marker. Stain intensity was scored as mild, moderate, or strong. The following formula was then applied to obtain a final score:


$$\begin{array}{l} \text{percentage of mildly positive cells}+2\text{x}\;\left(\text{percentage of moderately positive cells}\right)+3\text{x}\\ \left(\text{percentage of strongly positive cells}\right) \end{array}$$

Accordingly, the final score ranged from 0 to 300. Both HCC neoplastic cells and normal liver non-neoplastic cells were evaluated and scored.

### Data analysis and statistical calculations

Statistical analyses were performed using chi-square, Pearson correlation, Mann–Whitney nonparametric test, unpaired *t*-test with Welch’s correction, one-way ANOVA test with Tukey’s correction for multiple comparisons, or Kruskal–Wallis test for non-parametric values. Significance was taken at the 5% level. The normality of distribution was preliminarily assessed by using the Kolmogorov–Smirnov algorithm. For cell culture experiments, data in bar graphs represent means ± SEM and were obtained from the average data of at least three independent experiments. Kaplan–Meier curves of survival and time to recurrence were estimated using log-rank (Mantel–Cox) test.

## Results

### HRG deletion significantly reduces the development of MASH-associated hepatocellular carcinoma

**Figure 1:**
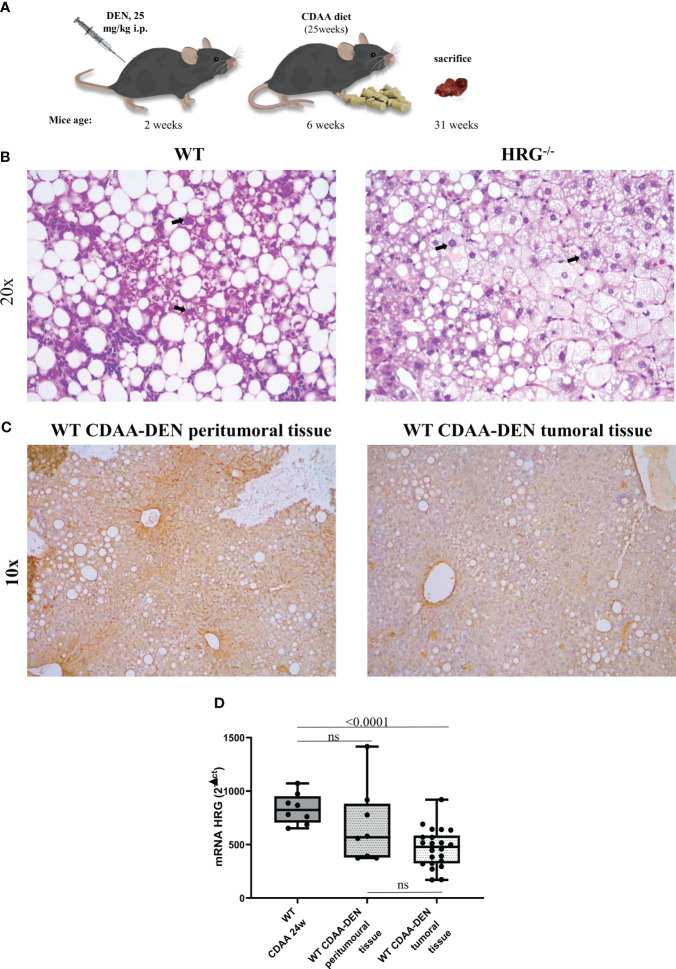
Experimental MASLD/MASH-related HCC: the DEN-CDAA murine model. Graphic representation of the rodent model of MASLD-associated hepatocarcinogenesis based on a single injection of diethyl-nitrosamine (DEN) at 2 weeks of age and the subsequent induction of steatohepatitis by the administration of a CDAA diet for 25 weeks **(A)**. Hematoxylin–eosin staining performed on paraffin-embedded HCC tumor masses from wild-type mice (WT) (*n* = 12) or from HRG *knock-out* mice (HRG^–/–^) (*n* = 11). IHC analysis for HRG performed on paraffin-embedded HCC tumor masses and peri-tumoral tissue from 12 wild-type mice (WT). The bold arrows indicate nuclear pleomorphism. Original magnification as indicated **(B, C)**. HRG expression analyzed by q-PCR in WT mice subjected to CDAA diet for 24 weeks or DEN-CDAA protocol **(D)**. The mRNA values are expressed as fold increase over control values after normalization to the TBP gene expression **(D)**. ns, not significant.

**Figure 2:**
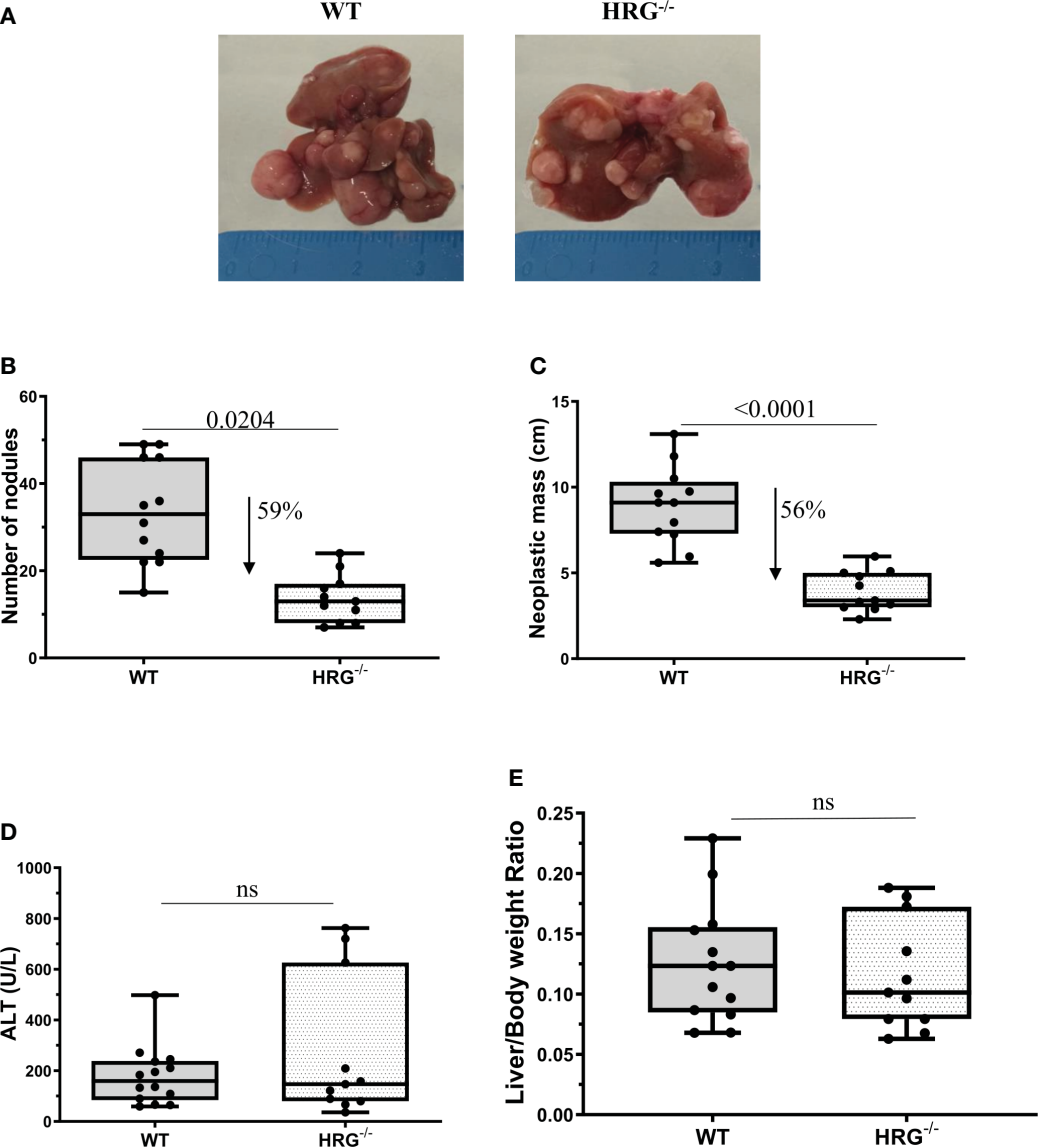
HRG deletion significantly affects the development of experimental liver tumors. Reduction of number and of neoplastic mass measured in HCC tumors from 12 wild-type mice (WT) or 11 from HRG *knock-out* mice (HRG^–/–^) **(A–C)**. The results are expressed as means ± SD. The boxes include the values within the 25th and 75th percentile, whereas the horizontal bars represent the medians. The extremities of the vertical bars (10th–90th percentile) comprise 80% of the values. Statistical differences were assessed by Student’s *t*-test **(B, C)**. Parenchymal injury estimated by measuring the circulating levels of alanine (ALT) is reported in WT and HRG^-/-^ mice **(D)**. Liver/body weight ratio measured in WT and HRG^-/-^ mice **(E)**. The results are expressed as means ± SD. The boxes include the values within the 25th and 75th percentile, whereas the horizontal bars represent the medians. The extremities of the vertical bars (10th–90th percentile) comprise 80% of the values. Statistical differences were assessed by Student’s *t*-test or Mann–Whitney test for non-parametric values **(D, E)**. ns, not significant.

To mechanistically investigate the role of HRG in the development of MASH-associated liver carcinogenesis, we employed HRG *knock-out* (HRG^-/-^) mice submitted to a murine protocol of MASLD-associated liver cancerogenesis based on a single injection of DEN at 2 weeks of age and, after 4 weeks of recovery, the subsequent induction of steatohepatitis by the administration of a choline-deficient L-amino acid–defined (CDAA) diet for a further 25 weeks (**Figure 1A**). In line with previous studies (42), the HCC cases arising in WT mice were morphologically resembling an Edmonson–Steiner G1/G2 grading and characterized by nuclear atypia, pleomorphism, and increased mitotic activity. In addition, like in human MASLD-related HCC (44), they showed diffuse fat accumulation in parenchymal cells (**Figure 1B**). Interestingly, the analysis of HRG transcripts and immunohistochemistry showed that HRG expression was significantly lower in tumor tissue compared with normal liver parenchyma (**Figures 1C, D**). However, in HCC-bearing animals, no significant differences were evident between tumoral and peri-tumoral areas (**Figures 1C, D**). The HCCs detected in HRG^-/-^ mice were morphologically similar to those in WT mice, particularly in terms of fat accumulation (**Figure 1B**). No significant changes in steatosis and lipid metabolism were detected, and neither likewise in the peritumoral tissue analyzed by morphological analysis together with tissue triglyceride analysis (**Supplementary Figures S2A, B**). Moreover, the transcriptional levels of some lipid metabolism-related genes such as adipose triglyceride lipase (ATGL), carnitine palmitoyltransferase 1A (CPT1A), medium-chain acyl-CoA dehydrogenase (MCAD), CD36 molecule (CD36), fatty acid synthase (FASN), fatty acid binding protein 4 (FABP4), sterol regulatory element binding transcription factor 1 (SREBP1), 3-hydroxy-3-methylglutaryl-CoA lyase (HMGCL), and 3-hydroxy-3-methylglutaryl-CoA synthase 2 (HMGCS2) did not change across the two experimental groups (**Supplementary Figure S2C**). However, HRG ablation reduced by 59% and 56%, respectively, the number and the size of the tumors compared with those in WT mice (**Figures 2A-–C**) despite the fact that no significant changes were found for either ALT plasma levels or liver weight/body weight ratio when comparing data from HRG^-/-^ and WT mice (**Figures 2D, E**).

### HRG deletion affects the development of experimental MASH-associated HCC by downregulating inflammatory response and fibrogenesis

**Figure 3:**
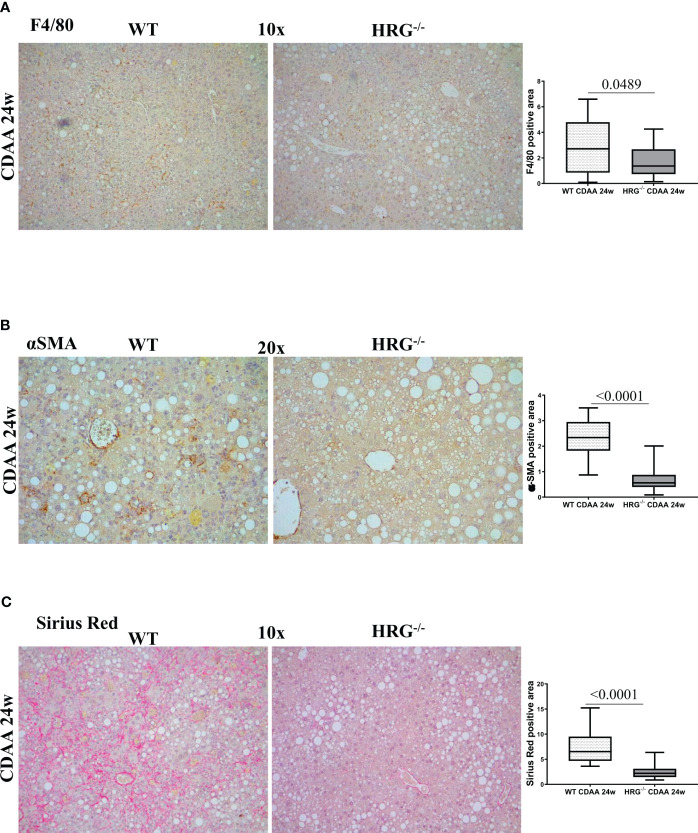
HRG deletion affects the development of experimental MASH by downregulating inflammatory response and fibrogenesis. IHC analysis for F4/80 **(A)**, αSMA **(B)** and Sirius Red staining **(C)** performed on paraffin-embedded liver samples from six wild-type mice (WT) or from nine HRG *knock-out* mice (HRG^–/–^) fed with CDAA diet for 24 weeks. ImageJ software analysis was performed to evaluate the amount of positivity. The data are means ± SD of 6/9 animals per group. The boxes include the values within the 25th and 75th percentile, whereas the horizontal bars represent the medians. The extremities of the vertical bars (10th–90th percentile) comprise 80% of the values. Statistical differences were assessed by unpaired parametric *t*-test or Mann–Whitney non-parametric *t*-test. Original magnification as indicated **(A–C)**.

A previous study reported that HRG acts as a pro-inflammatory mediator in MASLD and contributes to the fibrogenic progression of the disease (35), although these findings were derived from a MCD diet protocol (i.e., a relatively short protocol lacking features of the metabolic syndrome). In the present study, we found that HRG^-/-^ mice fed with a CDAA diet for 24 weeks, used as a model of advanced MASH (3), showed a significant reduction in the hepatic recruitment of F4/80^+^ macrophages as well as of α-smooth muscle (αSMA)-positive myofibroblast-like cells compared with the WT ones (**Figures 3A, B**). These events were accompanied by a significant reduction of collagen deposition (**Figure 3C**), confirming the contributing role of HRG in MASLD progression.

**Figure 4:**
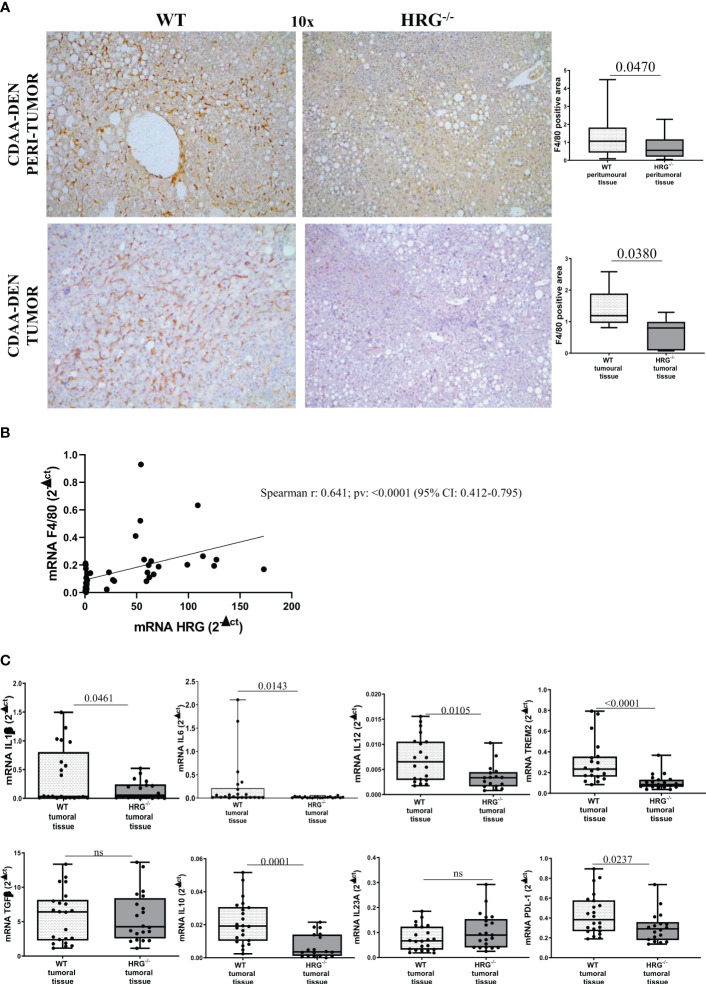
HRG deletion significantly affects inflammatory response. IHC analysis of F4/80 performed on paraffin-embedded HCC tumor masses and peri-tumoral tissue **(A)** from 12 wild-type mice (WT) or from 11 HRG *knock-out* mice (HRG^–/–^). ImageJ software analysis was performed to evaluate the amount of positivity. The data are means ± SD of 11/12 animals per group. The boxes include the values within the 25th and 75th percentile, whereas the horizontal bars represent the medians. The extremities of the vertical bars (10th–90th percentile) comprise 80% of the values. Statistical differences were assessed by unpaired parametric *t*-test or Mann–Whitney non-parametric *t*-test. Original magnification as indicated **(A)**. Relationship between HRG and F4/80 mRNA in HCCs from WT mice. The values represent the relative mRNA content. The correlation analysis was performed with Pearson *r* test **(B)**. qPCR analysis of IL1β, IL6, IL12, TREM2, TGFβ, IL10, IL23A, and PDL-1 **(C)** transcripts performed in HCCs from 12 WT mice or from 11 HRG^–/–^ mice. The mRNA values are expressed as fold increase over control values after normalization to the TBP gene expression. The results are expressed as means ± SD. The boxes include the values within the 25th and 75th percentile, whereas the horizontal bars represent the medians. The extremities of the vertical bars (10th–90th percentile) comprise 80% of the values. Statistical differences were assessed by Student’s *t*-test or Mann–Whitney test for non-parametric values **(C)**. ns, not significant.

We next investigated whether HRG deletion influenced inflammatory response during experimental carcinogenesis. F4/80 immunohistochemistry revealed that, compared to WT mice, HRG deletion appreciably lowered macrophage infiltration in both the tumoral and peritumoral tissues (**Figure 4A**). Consistently, we found a significant correlation between F4/80 and HRG transcripts among individual HCCs (**Figure 4B**). Furthermore, the expression of pro-inflammatory cytokines such as IL-1β, IL-6, and IL-12 was appreciably lower in HCCs from HRG^-/-^ mice than in those from WT mice (**Figure 4C**), whereas no significant difference was detected for TGFβ1 and IL23A mRNAs (**Figure 4C**). We also evidenced a significant decrease in transcript levels for IL-10, programmed death ligand 1 (PDL1), and triggering receptor expressed on myeloid cells 2 (TREM2), a surface receptor expressed by the so-called NASH-associated macrophages (NAMs) (45) as well as by HCC-associated macrophages (46) (**Figure 4C**).

To further investigate macrophage response to HRG, we evaluated the effect of human HRG supplementation in human monocytic THP1 cells, confirming the ability of HRG to upregulate the expression of pro-inflammatory cytokines (IL-1β, IL-6, and TNFα) (**Supplementary Figures S3A–C**) and IL-10 (**Supplementary Figure S3F**). Moreover, HRG also induced a significant upregulation of vascular endothelial growth factor A (VEGF-A) and IL23A (**Supplementary Figures S3D, E**).

**Figure 5:**
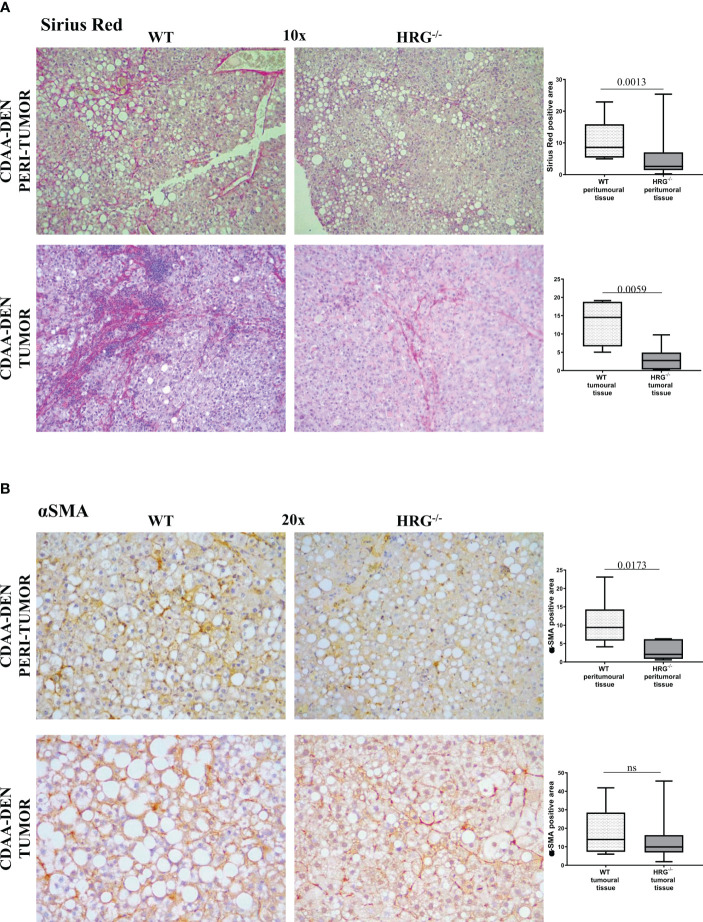
HRG deletion affects the development of experimental MASH-HCC by downregulating inflammatory response and fibrogenesis. Sirius Red staining **(A)** and IHC analysis for αSMA **(B)** performed on paraffin-embedded HCC tumor masses and peri-tumoral tissue from 12 wild-type mice (WT) or from 11 HRG *knock-out* mice (HRG^–/–^). ImageJ software analysis was performed to evaluate the amount of positivity. The data are means ± SD of 11/12 animals per group. The boxes include the values within the 25th and 75th percentile, whereas the horizontal bars represent the medians. The extremities of the vertical bars (10th–90th percentile) comprise 80% of the values. Statistical differences were assessed by unpaired parametric *t*-test or Mann–Whitney non-parametric *t*-test. Original magnification as indicated **(A, B)**. ns, not significant.

**Figure 6:**
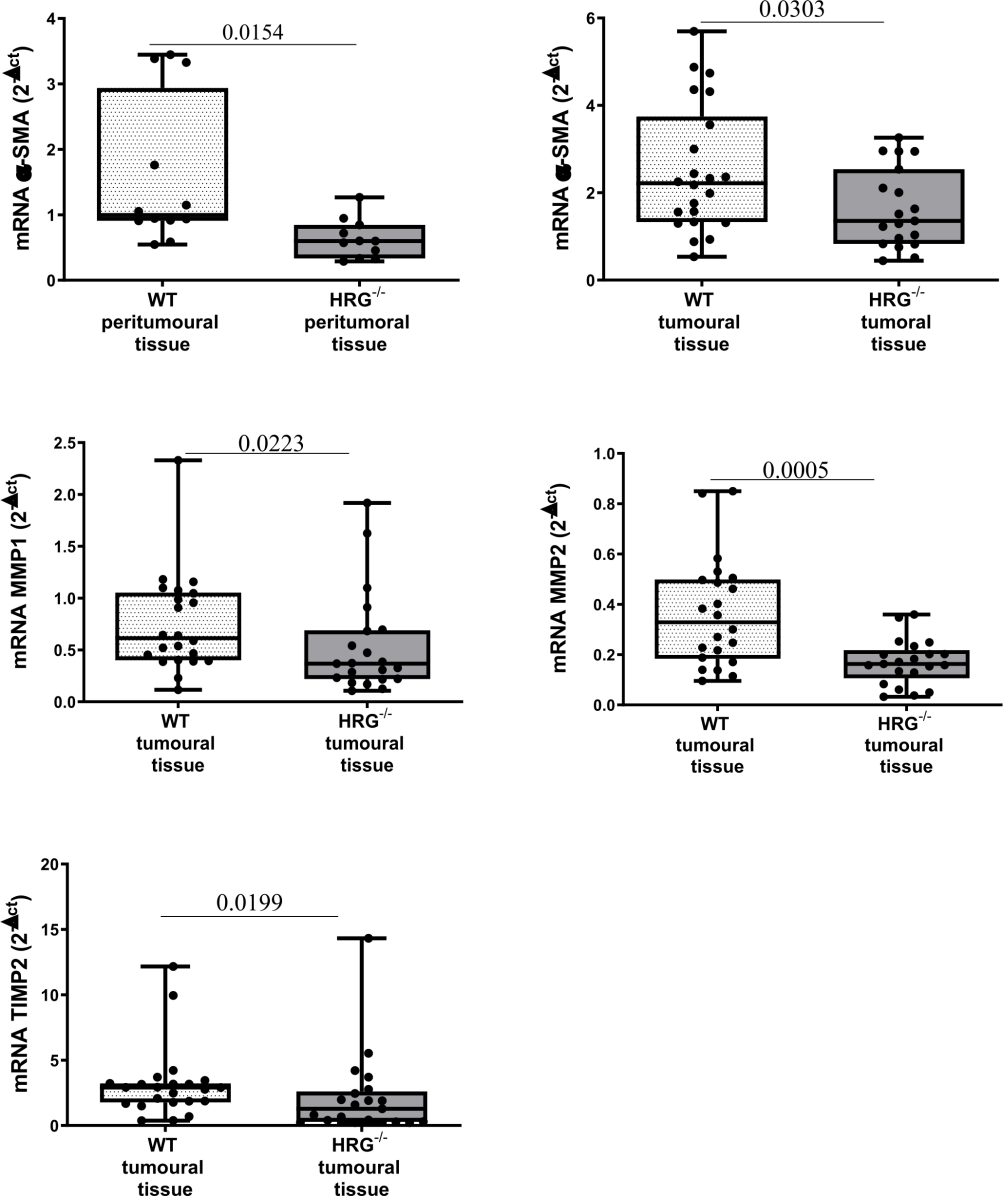
HRG deletion affects the development of experimental MASH-HCC by downregulating fibrogenesis. qPCR analysis of αSMA, MMP1, MMP2, and TIMP2 transcripts performed in HCC tumor masses or peri-tumoral tissue from 12 WT mice or from 11 HRG^–/–^ mice. The mRNA values are expressed as fold increase over control values after normalization to the TBP gene expression. The results are expressed as means ± SD. The boxes include the values within the 25th and 75th percentile, whereas the horizontal bars represent the medians. The extremities of the vertical bars (10th–90th percentile) comprise 80% of the values. Statistical differences were assessed by Student’s *t*-test or Mann–Whitney test for non-parametric values.

In addition to the effect on inflammatory response, collagen Sirius Red staining evidenced that HRG deletion significantly affected extracellular matrix deposition in both tumoral and peritumoral tissues (**Figure 5A**). In line with this observation, immunohistochemical analysis highlighted a significant decrease of αSMA^+^ myofibroblast-like cells in peritumoral areas but not in HCC nodules of HRG^-/-^ mice (**Figure 5B**); α-SMA transcripts were lowered in both peritumoral tissue and tumors from HRG^-/-^ mice. Conversely, HRG deletion affected MMP1, MMP2, and TIMP2 expression only in tumoral tissue (**Figure 6**).

### HRG deletion affects angiogenesis and apoptosis but not proliferation

**Figure 7:**
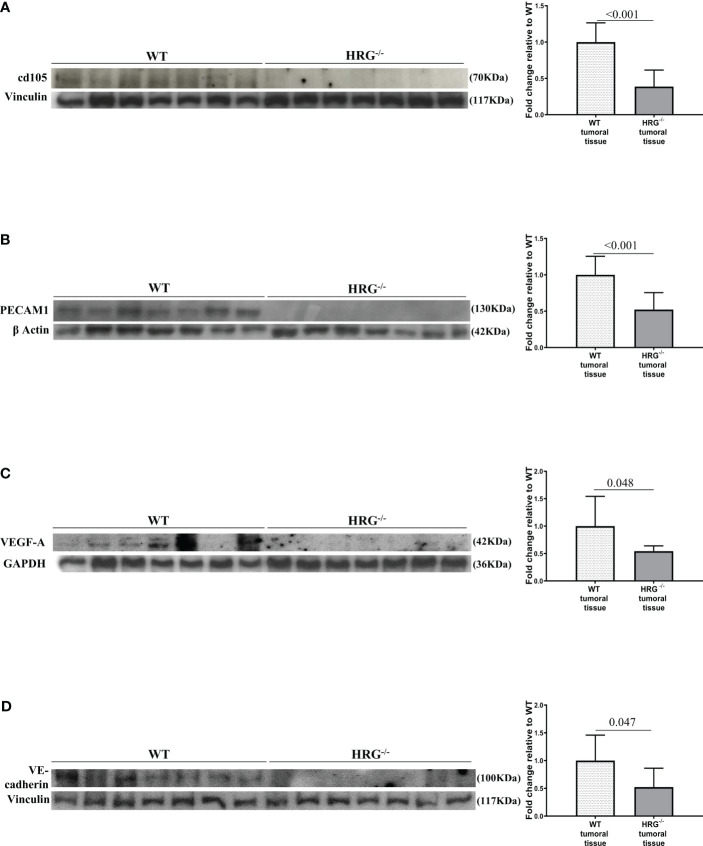
HRG deletion impact on HCC angiogenesis. WB analysis for cd105 **(A)**, PECAM1 **(B)**, VEGF-A **(C)**, and VE-cadherin **(D)** performed in HCCs from wild-type mice (WT) or from HRG *knock-out* mice (HRG^–/–^). BIORAD Quantity One software was used to perform the densitometric analysis (data are expressed as fold change relative to the normalized WT expression). Equal loading was evaluated by re-probing membranes for vinculin **(A, D)**, β-actin **(B)**, and GAPDH **(C)**. Statistical differences were assessed by Student’s *t*-test or Mann–Whitney test for non-parametric values.

**Figure 8:**
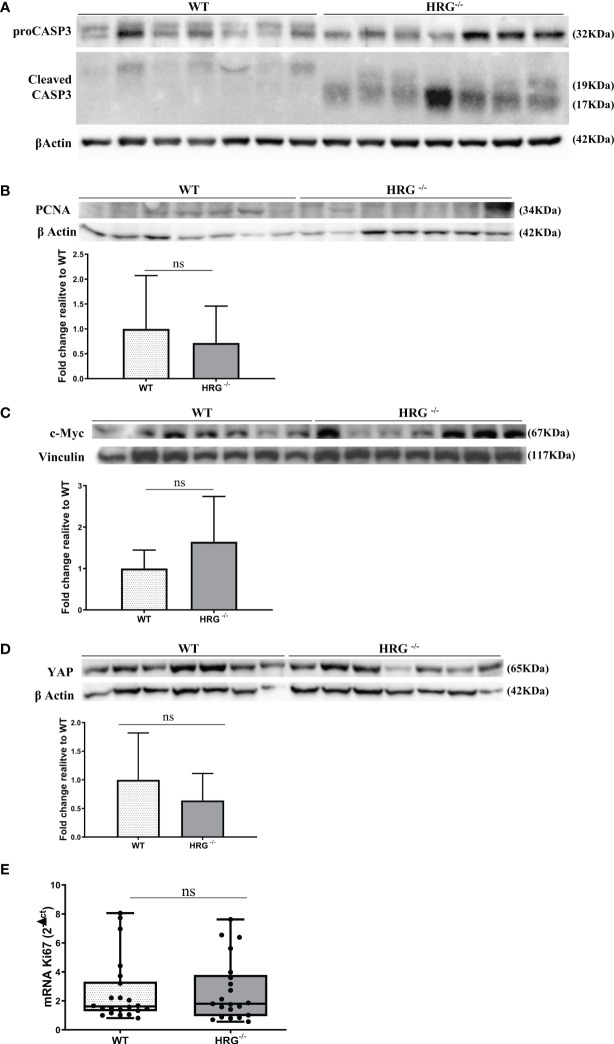
HRG deletion affects apoptosis but not proliferation. WB analysis for proCASP3 and cleaved CASP3 **(A)**, PCNA **(B)**, c-MYC **(C)**, and YAP **(D)** performed in HCCs from wild-type mice (WT) or from HRG *knock-out* mice (HRG^–/–^). BIORAD Quantity One software was used to perform the densitometric analysis (data are expressed as fold change relative to the normalized WT expression). Equal loading was evaluated by re-probing membranes for β-actin **(A, B, D)** or vinculin **(C)**. Statistical differences were assessed by Student’s *t*-test or Mann–Whitney test for non-parametric values **(A–D)**. qPCR analysis of Ki67 transcripts performed in HCC tumor masses from 12 WT mice or from 11 HRG^–/–^ mice **(E)**. The mRNA values are expressed as fold increase over control values after normalization to the TBP gene expression. The results are expressed as means ± SD. The boxes include the values within the 25th and 75th percentile, whereas the horizontal bars represent the medians. The extremities of the vertical bars (10th–90th percentile) comprise 80% of the values. Statistical differences were assessed by Student’s *t*-test or Mann–Whitney test for non-parametric values **(E)**. ns, not significant.

From the observation that HRG stimulates macrophage VEGF-A expression, we moved to analyze the effects of HRG on the angiogenic process. In this setting, we found a drastic decrease in CD105, PECAM1, VEGF-A, and VE-cadherin protein expression in the tumors obtained from HRG^-/-^ mice (**Figures 7A–D**). HRG deletion also induced a higher apoptotic rate within HCC nodules as testified by an increase in the cleavage of CASP3 (**Figure 8A**). Enhanced cell death was not accompanied by a concomitant reduction of the proliferative rate of tumor cells since no significant difference was found in the protein levels of proliferation markers such as PCNA, c-MYC, YAP, and transcript levels of Ki67 (**Figures 8B–E**). Overall, these data indicate that the reduction in the HCC number and size in HRG ^-/-^ mice was likely related to an impairment of angiogenesis and an increase in the rate of cancer cell apoptosis.

### HRG is detectable in human HCC of mixed etiology and associated with survival

**Figure 9:**
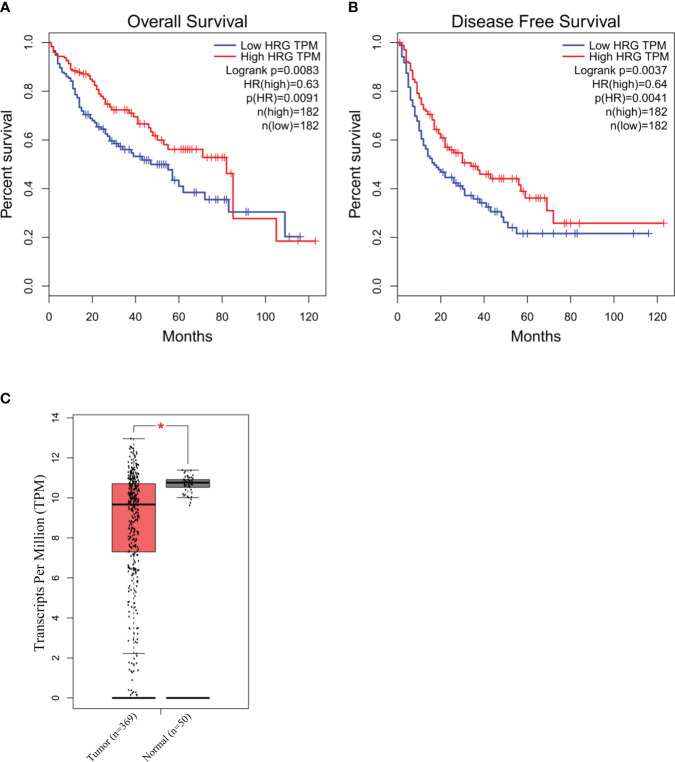
HRG in human HCC of mixed etiology. Survival and disease-free survival (log rank Mantel–Cox test) in high- vs. low-HRG-expressing HCC patients in the TCGA-LIHC cohort accessed using GEPIA webserver **(A, B)**. GEPIA analysis of HRG gene expression in the non-tumoral (*n* = 50) areas and in the tumor samples (*n* = 369) from the TCGA-LIHC cohort. *p-value <0.0001 **(C)**.

To explore the possible clinical significance of HRG in HCC pathogenesis, we initially took advantage of The Cancer Genome Atlas (TCGA) database related to human HCC. The TCGA database was accessed using GEPIA webserver to retrieve HRG gene expression in a cohort of patients (LIHC) with mixed etiology HCCs. From this preliminary approach, the log rank (Mantel–Cox) test demonstrated that patients with higher levels of HRG had a hazards ratio <1 for overall survival and disease-free survival, a feature that may suggest an apparent protective role for HRG in LIHC patients (**Figures 9A, B**). Interestingly, the GEPIA analysis also revealed that HRG expression was significantly higher in the non-tumoral areas compared with the tumor tissue (**Figure 9C**).

### HRG clinical significance in a cohort of MASH patients carrying HCC or not

**Figure 10:**
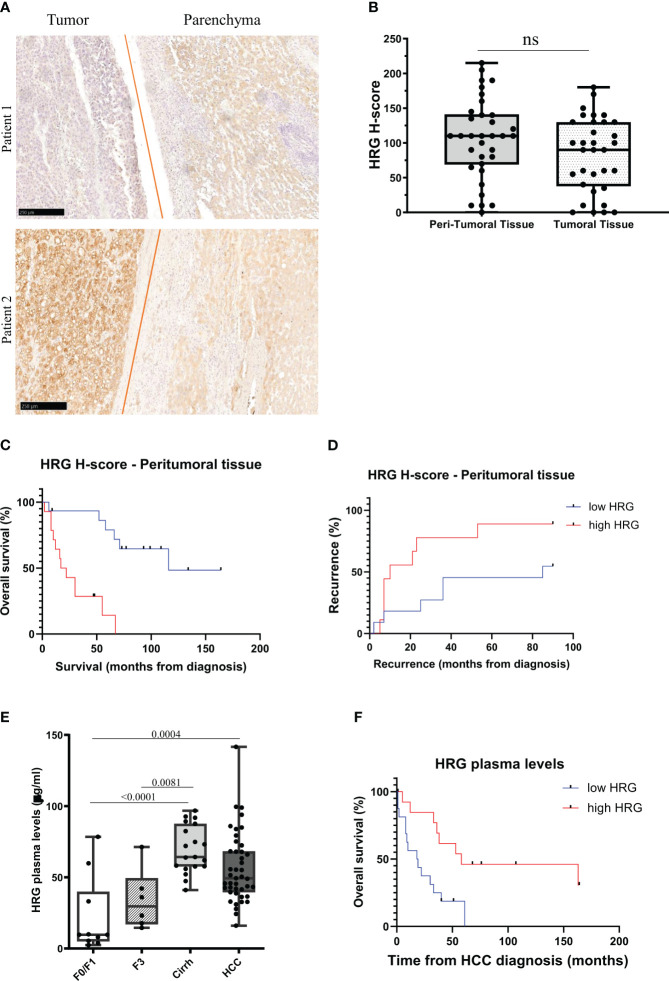
HRG protein expression in MASLD/MASH-related HCC patients. IHC analysis of HRG performed on paraffin-embedded human liver specimens from MASLD/MASH-related HCC patients (*n* = 37, grades G2 to G3). Original magnification as indicated **(A)**. HRG expression was semi-quantitatively scored blinded by a pathologist by means of histological score (H-score) **(B)**. Kaplan–Meier curves of overall survival **(C)** and recurrence **(D)** according to HRG peritumoral H-score in MASLD/MASH-related HCC patients **(C, D)**. Statistical analysis was performed using log-rank (Mantel–Cox) test **(C, D)**. Plasma concentration of HRG measured in a cohort of patients (*N* = 78) with different stages of disease progression including 10 patients with a F0/F1 score, 25 patients with a F3/F4 score (six F3 and 19 F4) and 43 MASLD/MASH patients carrying HCC. Cirrh, cirrhotic liver; F, fibrosis score **(E)**. Kaplan–Meier curves of overall survival according to HRG plasma levels in MASLD/MASH-related HCC patients **(F)**. Statistical analysis was performed using log-rank (Mantel–Cox) test **(F)**. ns, not significant.

From the abovementioned observations, we investigated in more detail HRG involvement in human MASLD/MASH-related HCCs by performing an immune-histochemical analysis of HRG expression in specimens obtained from a cohort of 37 MASH-related HCC patients (see **Table 1** for clinical and biochemical characterization of patients), observing a large interindividual variability in HRG staining in both the tumoral and peritumoral tissues (**Figure 10A**). As evaluated by histological score (H-score), HRG was moderately to highly expressed (H-score ≥100) in the tumoral tissue of 16 out 37 patients (43.2%) and in the peri-tumoral tissue of 20 out 37 (56.8%) without significant differences in HRG expression between tumoral and peri-tumoral tissues (**Figures 10A, B**). Interestingly, patients with high peritumoral HRG expression showed a higher HCC recurrence (Mantel–Cox log-rank 4.3, *p* = 0.037) and a worst overall survival (Mantel–Cox log-rank 15.6, *p* < 0.0001) (**Figures 10C, D**). The same trend was also observed by considering HRG detectable in tumor parenchyma (**Supplementary Figures S4A, B**), but due to the large interindividual variability, the differences here did not reach statistical significance.

These results and the data emerging from experimental studies prompted us to evaluate whether circulating HRG might be used as a marker for MASH evolution to HCC. To this aim, we investigated a cohort of 78 MASLD/MASH patients at different disease stages (see **Table 2** for the clinical and biochemical characterization of patients), including 10 patients with a F0/F1 score, 25 subjects with advanced fibrosis (six F3) or cirrhosis (19 cirrh), and 43 subjects with MASLD/MASH-related HCCs. In these subjects, we observed a significant increase in HRG plasma levels during the disease progression, with significant differences between patients at advanced stages (cirrhotic) compared to those at early stages (F0/F1) (**Figure 10E**). Although HCC patients displayed HRG plasma levels higher than those in non-cirrhotic patients, the circulating HRG was lower in HCC than in cirrhotic subjects (**Figure 10E**). Furthermore, plasma HRG among HCC patients showed a large interindividual variability which negatively correlated (*r* = 0.42; *p* = 0.001) with ALBI (albumin–bilirubin) score for HCC, a specific prognostic index used for HCC patients (47), suggesting that HRG reduction during the progression from cirrhosis to HCC likely reflects the worsening of the hepatic functions. Consistently, in this cohort of MASH-related HCC patients, lower HRG plasma levels associated with poorer survival (Mantel–Cox log-rank 7.8; *p* = 0.005; **Figure 10F**).

## Discussion

HRG has been previously reported to be upregulated in CLDs of different etiologies, specifically in patients affected by chronic HCV infection or MASLD/MASH (35, 36). In particular, during MASLD/MASH, HIF2α activation stimulates hepatocytes to produce HRG (36), which acts as a pro-inflammatory and profibrogenic mediator (35, 36). However, so far, the role of HRG in MASH-related hepatocarcinogenesis has not been investigated.

To address this issue, we have used HRG^-/-^ mice submitted to a well-established protocol of MASH-related liver carcinogenesis (42). HRG-deficient mice are well suited for our purpose because HRG is expressed mainly by hepatocytes in both humans and rodents (30, 31, 44), and HRG^-/-^ mice have already been used to investigate HRG’s role in MASH progression (35). In these settings, we found that HRG deletion leads to a very significant decrease in both the number and size of HCC nodules in the absence of any significant change in fat accumulation or proliferative indexes. These results differ from previous observations showing an extensive growth and metastasization of fibrosarcoma and pancreatic carcinoma transplanted in HRG^-/-^ mice (37). In the same study, the authors provide evidence that macrophages are a direct HRG target, concluding that HRG deletion may favor cancer cell growth and metastasis by modulating macrophage phenotypes with the stimulation of pro-angiogenic and immunosuppressive functions (37). Along these lines, it should be noted that, in addition to the well-established role of macrophages, either resident or continuously recruited from peripheral blood in sustaining the progression of MASH, there is overwhelming evidence that macrophages are also critical for HCC development and progression (26–28). In MASH, pro-inflammatory macrophages exacerbate liver inflammation and oxidative stress by producing inflammatory cytokines, leading to hepatocyte injury and advancing liver fibrosis (48). In HCC, pro-inflammatory macrophages foster tumor growth and progression by stimulating angiogenesis and supporting tumor vascularization and growth. Moreover, they prevent antitumor immune responses, establishing a microenvironment favorable for tumor progression (49–51). In our hands, the lack of HRG causes a marked lowering of macrophage infiltration in MASH-related HCCs along with a reduction in the expression of pro-inflammatory cytokines (IL-1β, IL-6, and IL-12) as well as of IL-10 and TREM2, a marker of HCC-associated macrophages (45, 46). Interestingly, *in vitro* experiments using THP1 human monocytic cell line confirm that the supplementation with human purified HRG effectively stimulated the expression of pro-inflammatory cytokines and IL-10. In the same experiments, we also observed that HRG promoted VEGFA expression, supporting *in vivo* data showing that HRG deletion severely affects the production of several pro-angiogenic markers in MASH-related HCCs. Such a decrease in angiogenesis along with an enhanced rate of cell apoptosis can explain the reduction in the number and size of HCC nodules detected in HRG^-/-^ mice. It is noteworthy that HRG effects on HCC development do not involve changes in extracellular matrix since the presence of αSMA-positive myofibroblast-like cells in MASH-related HCCs from HRG^-/-^ is comparable with that in WT mice. Conversely, according to previous observations (35, 36), the lack of HRG reduces liver collagen deposition and the prevalence of α-SMA-positive cells in peritumoral tissue in WT mice. These data altogether suggest the possibility that during MASH evolution to HCC, hepatic HRG production might sustain a specific population of tumor-associated macrophages capable of stimulating an inflammatory response along with angiogenesis and critically supporting cancer cell survival. Such a hypothesis is consistent with our previous observations that hepatocyte conditional deletion of HIF2α significantly prevents HRG production by MASH livers (36) as well as MASH-related liver carcinogenesis (42).

The translational potential of the data obtained in rodents is supported by the analysis of circulating HRG in a cohort of MASLD/MASH patients at different disease stages, showing that HRG plasma levels not only increase during disease progression but are also elevated in a large fraction of the subjects carrying HCCs. The HRG values in the latter are, however, very variable and likely reflecting the impairment of liver functions in HCC-bearing subjects as evidenced by the negative correlation between circulating HRG and ALBI (albumin–bilirubin) score for HCC. A more specific analysis using immunohistochemistry confirmed a large interindividual variability in production by human HCCs. As observed in rodents, human MASH-related HCCs showed a tendency for a lower HRG production by cancer cells compared to the peritumoral liver parenchyma. Such a difference was confirmed by HRG gene expression in HCCs of mixed etiology extracted from The Cancer Genome Atlas (TCGA) database, suggesting that, despite HRG being critical for MASLD/MASH progression and for the onset of HCCs, it likely loses its importance in the advanced phase of liver cancer progression most likely because of the antiproliferative activity exerted in liver cancer cells (38, 39). On the same vein, Deuschle and coworkers have reported that HRG transcripts are significantly lower in human HCC than normal or non-tumoral liver, with a progressive decrease according to the tumor stage (40). Similarly, Cai and colleagues (41) have observed HRG downregulation in 28 of 31 human HCCs caused by aflatoxin B1.

Despite the data originating from TCGA database indicating a better survival rate in the subjects with HRG-expressing HCCs, the analysis of a small cohort of MASH-related HCCs show that high HRG staining of peritumoral tissue predicts an increase in tumor recurrence and a poor survival rate, thus supporting the experimental findings.

These inconsistencies can be explained by the possibly different role played by HRG in the pathogenesis of MASLD/MASH-related HCCs with respect to those from other etiologies. In the former, HRG is critical for supporting inflammation during both MASH progression to cancerogenesis and the presence of tumor-associated macrophages. However, in other settings, HRG can favor angiogenesis by interfering with trombospondin 1 (TSP1). Indeed TSP1 binding to CD36 has been reported to represent a potent antiangiogenic stimulus (31, 52–54) and HRG can act as a soluble decoy receptor for TSP-1 through a domain analogous to the CLESH domain of CD36 (55, 56). Accordingly, CD36 deletion favors the growth of Lewis lung carcinoma (LL2) and B16F1 melanoma tumor cell implants, while the growth of the same tumors is impaired in syngeneic HRG null mice (57). Collectively, the data obtained in the murine model indicate that HRG plays a significant role in MASLD/MASH progression to HCC by supporting a specific population of tumor-associated macrophages with pro-inflammatory response and pro-angiogenetic capabilities which critically support cancer cell survival. Further clinical investigations are needed to better clarify the actual significance of HRG expression in the evolution of human MASLD/MASH-related HCC and to determine its prognostic significance.

## Data availability statement

The original contributions presented in the study are included in the article/**Supplementary Material**, further inquiries can be directed to the corresponding author/s.

## Ethics statement

The studies involving humans were approved by ethics committee of the Azienda Ospedaliera Universitaria Città della Salute, Torino, Italy. The studies were conducted in accordance with the local legislation and institutional requirements. The participants provided their written informed consent to participate in this study. The animal study was approved by Animal Ethical Committee of University Amedeo Avogadro of East Piedmont, Novara, Italy and Animal Investigation Committee of the Italian Ministry of Health. The study was conducted in accordance with the local legislation and institutional requirements.

## Author contributions

BF: Conceptualization, Data curation, Formal analysis, Investigation, Methodology, Project administration, Software, Validation, Writing – original draft, Writing – review & editing. SS: Conceptualization, Data curation, Formal analysis, Software, Validation, Writing – original draft, Writing – review & editing. SC: Data curation, Formal analysis, Writing – review & editing. CR: Investigation, Methodology, Writing – review & editing. MM: Investigation, Methodology, Writing – review & editing. AC: Investigation, Methodology, Writing – review & editing. CB: Investigation, Methodology, Writing – review & editing. EN: Investigation, Methodology, Writing – review & editing. FP: Investigation, Methodology, Writing – review & editing. NR: Investigation, Methodology, Writing – review & editing. AP: Investigation, Methodology, Writing – review & editing. AG: Investigation, Methodology, Writing – review & editing. EB: Conceptualization, Data curation, Formal analysis, Funding acquisition, Resources, Software, Validation, Writing – original draft, Writing – review & editing. FT: Conceptualization, Writing – review & editing. EA: Conceptualization, Data curation, Formal analysis, Project administration, Software, Validation, Writing – original draft, Writing – review & editing. MP: Conceptualization, Data curation, Formal analysis, Funding acquisition, Project administration, Resources, Validation, Writing – original draft, Writing – review & editing.
