# Supplementary material for: Histidine-rich glycoprotein in metabolic dysfunction-associated steatohepatitis-related disease progression and liver carcinogenesis
Source: Front Immunol. 2024 Feb 26;15:1342404. doi: 10.3389/fimmu.2024.1342404 (PMC10925642; doi:10.3389/fimmu.2024.1342404)
Supplement: Supplementary file 1 [file DataSheet_1.pdf]

Supplementary Material

1 Supplementary Figures

Supplementary Figure 1

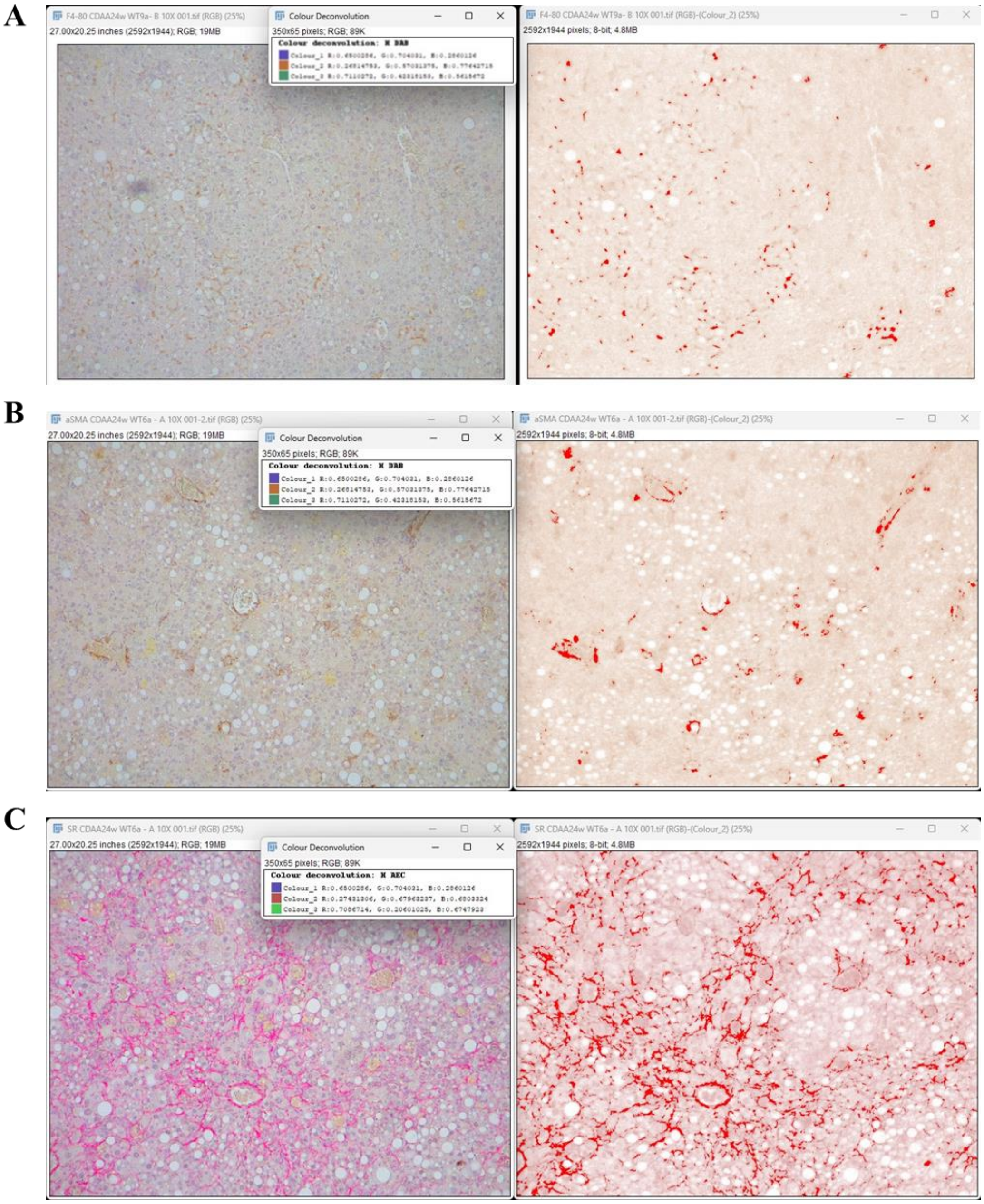

Supplementary Figure 1. ImageJ software application's accuracy in detecting the mentioned markers. Representative figure of the raw IHC image (left panels) and ImageJ threshold application into DAB staining for F4/80 and α-SMA protein expression (**A, B**) or Sirius Red staining (**C**) within liver tissue (right panels).

Supplementary Figure 2

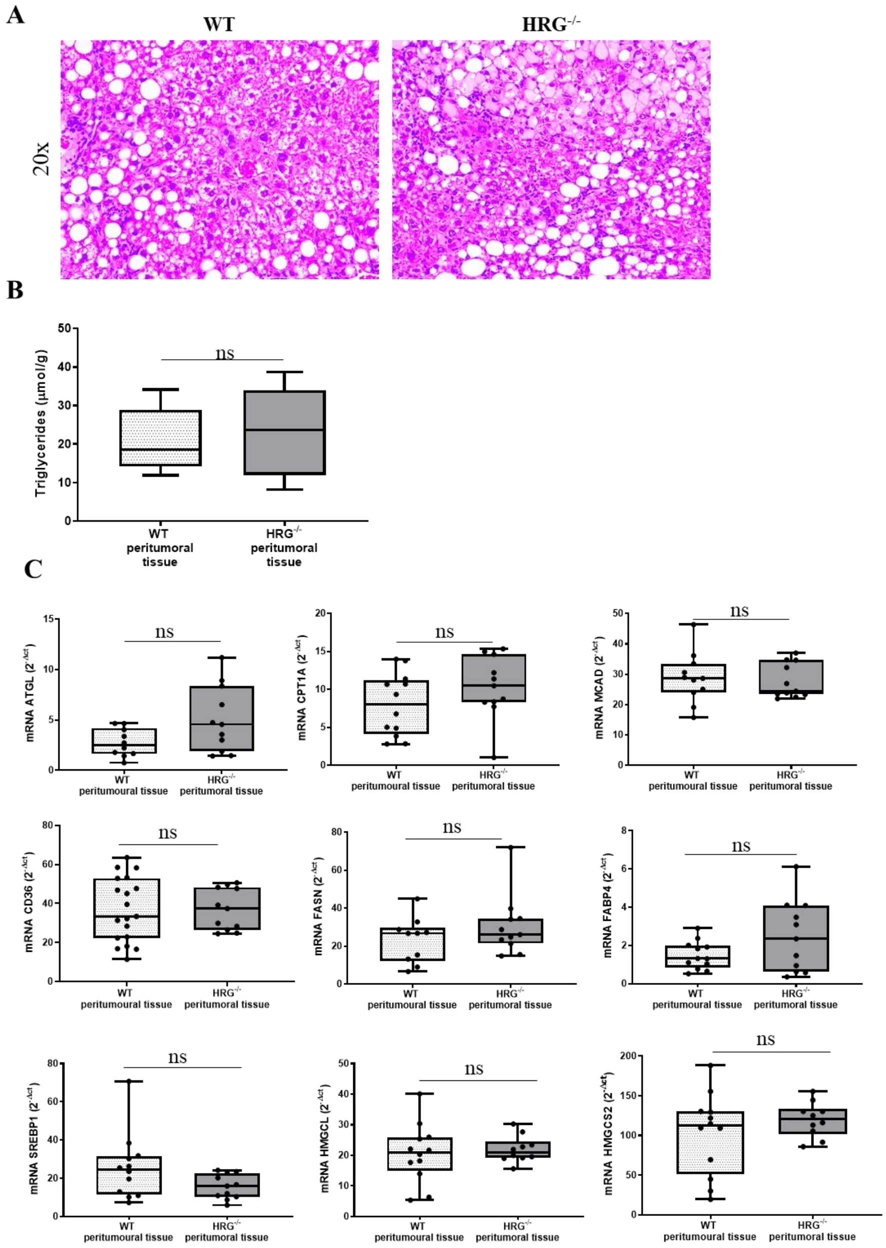

Supplementary Figure 2. Analysis of lipid metabolism in WT and HRG-/- mice. (**A**). Hematoxylin Eosin staining performed on paraffin-embedded peritumoral liver samples from wild type mice (WT) (n = 12) or from HRG knock-out mice (HRG–/–) (n = 11). Original magnification as indicated. (**B**). Tissue triglycerides concentration determined by Triglyceride Assay Kit - Colorimetric in peritumoral tissue samples from WT or from HRG–/– mice. (**C**) qPCR analysis of ATGL, CPT1A, MCAD, CD36, FASN, FABP4, SREBP1, HMGCL, HMGCS2 transcripts performed in peritumoral tissue from 12 WT mice or from 11 HRG–/– mice. The mRNA values are expressed as fold increase over control values after normalization to the TBP gene expression. Results are expressed as means ± SD. Boxes include the values within 25th and 75th percentile, whereas horizontal bars represent the medians. The extremities of the vertical bars (10th-90th percentile) comprise 80% of the values. Statistical differences were assessed by Student’s t test or Mann-Whitney test for non-parametric values.

Supplementary Figure 3

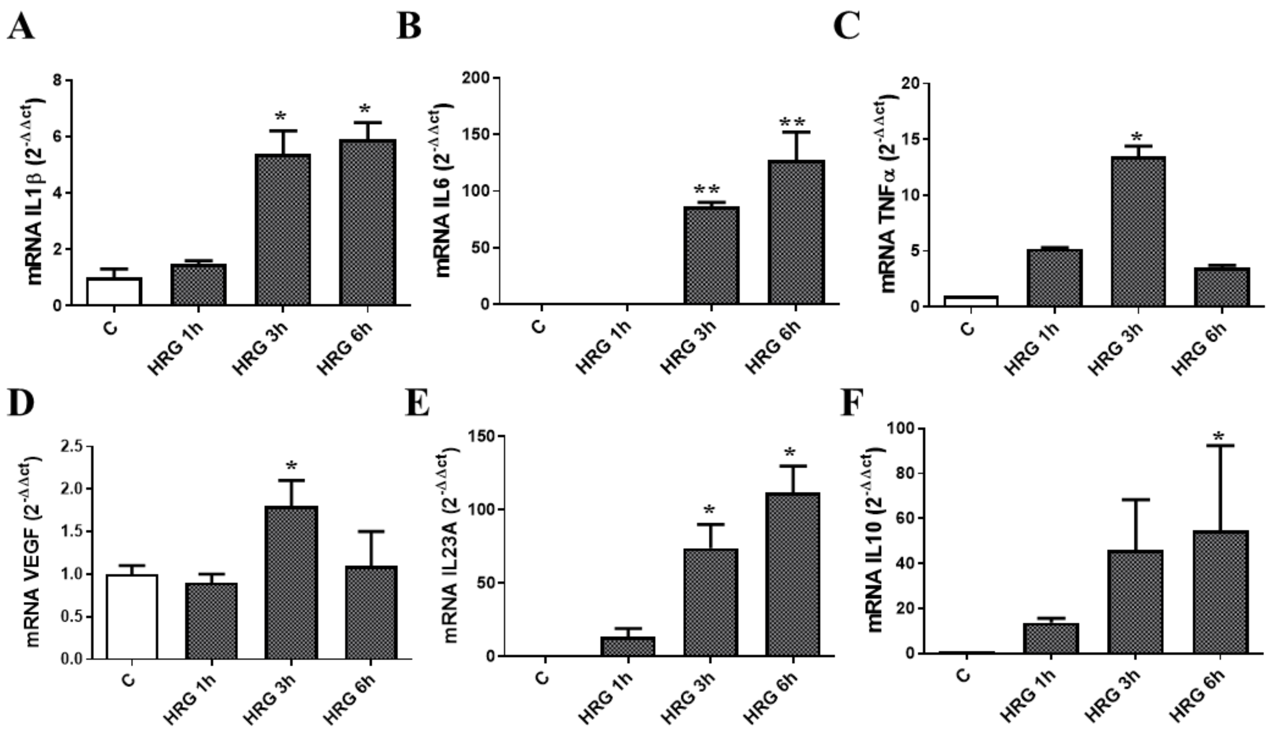

Supplementary Figure 3. HRG significantly affects the inflammatory response. q-PCR analysis of IL1β (**A**), IL6 (**B**), TNFα (**C**), VEGF (**D**), IL23A (**E**), IL10 (**F**) transcripts in THP-1 cells treated with 80μg/ml purified HRG at different time point. Data in graphs are expressed as means ± SEM. Statistical differences were assessed by one-way ANOVA test with Tukey's correction for multiple comparisons or Kruskal-Wallis test for non-parametric values (**A-F**).

Supplementary Figure 4

A

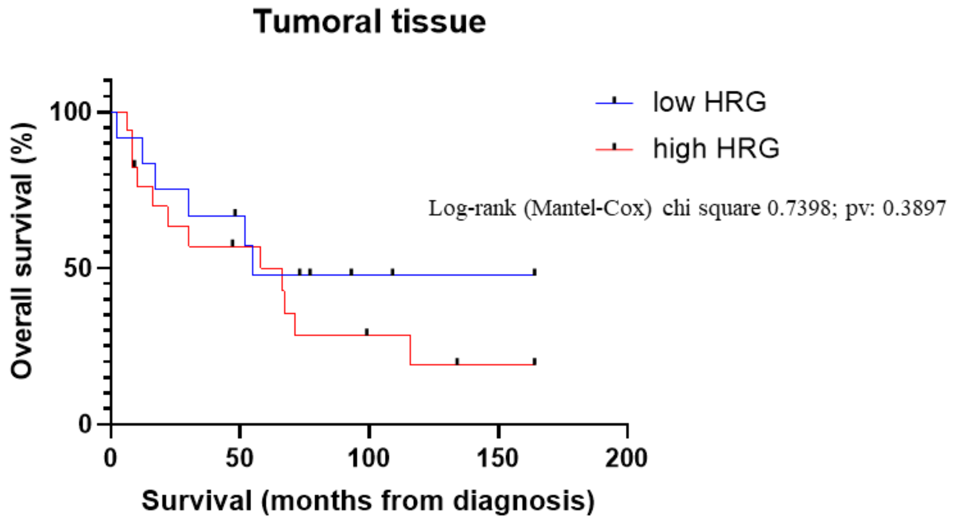

B

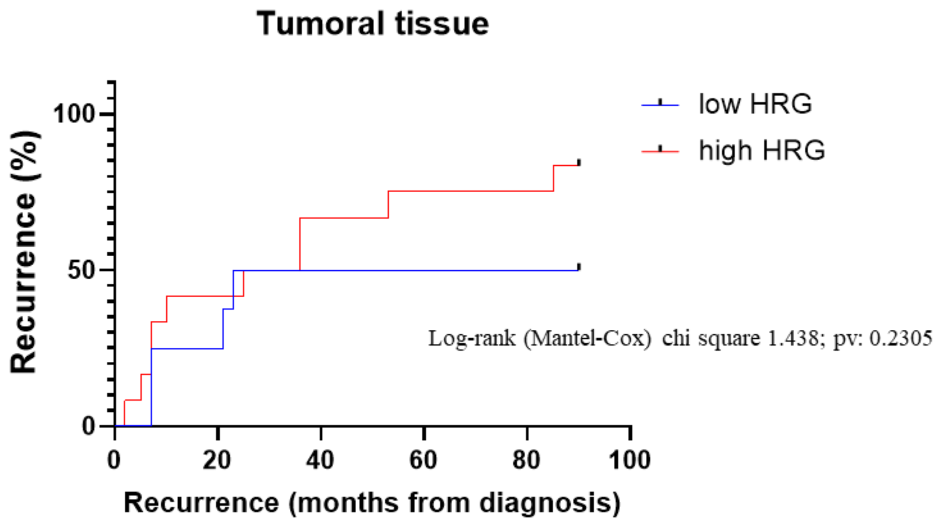

Supplementary Figure 4. Kaplan–Meier curves of overall survival (**A**) and recurrence (**B**) according to HRG tumoral H-score in MASLD/MASH-related HCC patients. Statistical analysis was performed using log-rank (Mantel–Cox) test (**A,B**).
